# Clinical Bedside Benchmarking Test for Measuring the Total Hemoglobin Concentration

**DOI:** 10.3390/healthcare13101102

**Published:** 2025-05-09

**Authors:** Elena Stawschenko, Stefan S. Niemuth, Benjamin Kern, Berit Bode, Frank Dörries, Christoph Marquetand, Kristina Kusche-Vihrog, Hartmut Gehring, Philipp Wegerich

**Affiliations:** 1 Department of Anaesthesiology and Intensive Care Medicine, University Medical Center Schleswig-Holstein, Campus Luebeck, 23538 Luebeck, Germany; elena.stawschenko@uksh.de (E.S.); berit.bode@uksh.de (B.B.); h.gehring@alumni.uni-luebeck.de (H.G.); 2 Medical Sensors and Devices Laboratory, Luebeck University of Applied Sciences, 23562 Luebeck, Germany; benjamin.kern@th-luebeck.de; 3 Northern Scientific Tec & Integration GmbH, Kollaustr. 11-13, 22525 Hamburg, Germany; frank.doerries@email.de; 4 Department of Cardiology, Angiology and Intensive Care Medicine, University Medical Center Schleswig-Holstein, Campus Luebeck, 23538 Luebeck, Germany; 5 Institute of Physiology, University of Luebeck, 23562 Luebeck, Germany; kristina.kuschevihrog@uni-luebeck.de; 6 Department of Anaesthesiology and Intensive Care Medicine, University of Luebeck, 23562 Luebeck, Germany; 7 Institute of Biomedical Engineering, University of Luebeck, 23562 Luebeck, Germany

**Keywords:** hemoglobin, accuracy, concentration, prediction interval, machine learning, intensive care unit

## Abstract

Objective: Accurate total hemoglobin concentration (ctHb) measurement is critical for clinical decision-making, particularly in acute care, where immediate therapeutic decisions are required. This study evaluated previously established laboratory-based accuracy criteria for ctHb measurements in routine clinical practice at an interdisciplinary operative intensive care unit (IO-ICU), and with particular attention to significantly reduced hemoglobin concentrations. Method: Remaining blood from blood gas analysis (BGA) cuvettes was collected directly at the ICU bedside. From these initial samples, three clinically relevant measurement scenarios were established: direct bedside measurement (Group 01), elevated ctHb levels (Group 02), and lowered ctHb concentrations below 9 g/dl (Group 03). The samples were analyzed using the GEM 4000, GEM 5000 (Werfen GmbH, Muenchen, Germany), ABL90 Flex plus (Radiometer GmbH, Krefeld, Germany), HemoCue Hb 201+, and XN 9000/9100 (Sysmex Deutschland GmbH, Norderstedt, Germany) automatic hematology analyzers. Since each measurement device inherently possesses systematic deviations, no single analyzer was defined as an absolute reference. Instead, the mean value across all tested measurement systems was utilized as a best-fit reference (REF) value. Results: A total of 120 data pairs from 40 ICU patients were analyzed using regression analyses, Bland and Altman (B&A) methods, and tolerance level analysis (TLA). The results demonstrated strong concordance among the evaluated measurement devices across the examined ctHb spectrum (~1–18 g/dL). Moderate systematic deviations identified by B&A analysis were most pronounced at critically low ctHb levels (<6 g/dL). A key outcome was the determination of 95% prediction intervals (PIs), representing a quantifiable range of uncertainties for future bedside measurements. The PIs for Group 03 “low” were in the range of ±7% (relative difference) or ±0.38 g/dL (absolute difference). Conclusion: This study effectively translates previous laboratory findings into clinical practice, highlighting the practical utility of PIs to guide the accurate interpretation of bedside ctHb measurements under acute care conditions.

## 1. Introduction

The rapid and accurate measurement of direct hemoglobin concentration (ctHb) is essential in life-threatening situations [1,2,3]. This is particularly critical in acute hemorrhagic emergencies [4,5] and cases of severe intra- or postoperative blood loss [6,7]. When immediate therapeutic decisions are required, only a brief measurement by means of blood gas analysis or other alternative point-of-care testing (POCT) procedures is usually indicated [2]. As ctHb decreases, the criticality of the decision increases [8,9].

A previous study investigated the requirements for the measurement accuracy of clinical methods in the precise determination of ctHb in [g/dL] within the range of 3–18 g/dL [10]. The clinically used measurement devices demonstrated almost perfect compliance with these requirements, especially in the critical range below 9 g/dL. However, translating these laboratory-based results into direct clinical application presents further challenges.

First, to what extent can these results be extrapolated to a single measurement value obtained from a blood sample taken directly from a patient and measured once within the clinical workflow of an intensive care unit (ICU)? Preanalytical processing and handling by experienced ICU staff are relevant considerations here.

Second, generating a data pool with a significantly reduced ctHb of 1–9 g/dL requires recruitment directly from pooled blood samples of a single patient, measured in a standardized and clinically comparable manner, as described in the first point. Such low ctHb levels—particularly in the range < 6 g/dL—are rarely recorded in direct clinical management [8] and typically require rapid therapeutic intervention.

Third, the chosen study design enables the generation of a data pool not only for low ctHb, but also for the higher range of ctHb. These data also provide valuable insights into the measurement accuracy at this level.

Fourth, the determination of PIs for defined ctHb values will enable clinicians to reliably assess the accuracy of measurements taken at the patient’s bedside.

Why a PI? Data on the measurement accuracy of ctHb and derived variables, such as the confidence interval (CI), refer to past analysis, and—in the case of CI—to mean values. However, a PI projects future performance and can be directly applied to clinically measured individual values [11]. Although PIs are generally wider than CIs, they allow for verified applicability at the patient’s bedside if predefined clinical specifications (e.g., tolerance levels [12]) are met.

The objectives of this study address these challenges. The study protocol was designed to facilitate further analysis using samples collected for BGA from ICU patients before they were discarded. It should be emphasized that this plan included both initial direct measurements and the targeted inclusion of low ctHb values—an approach that cannot be standardized in routine clinical settings due to therapeutic constraints. Moreover, these data are not yet available in this context. Presenting the data in the form of PIs aligns with emerging trends in self-learning algorithms [13] and provides clinicians with a clear, reliable tool for interpreting bedside measurements [14].

## 2. Materials and Methods

### 2.1. Blood Gas Samples

For the present study, blood samples were collected during the inpatient procedure and analyzed with a BGA device at the IO-ICU. As only a small volume was removed from the BGA cuvette (approx. 150 µL), a sufficient amount of blood remained for further laboratory investigations. The resulting data were then processed and documented in an anonymized form. The study protocol was approved by the local ethics committee of the University of Lübeck (registration number 20–224) and conducted in accordance with the Declaration of Helsinki Ethical Principles and Good Clinical Practices.

The standard cuvettes used for blood gas analysis were blood gas Monovettes^®^ (Sarstedt, AG & Co. Kg, Nümbrecht, Germany), prepared with dry calcium-balanced lithium heparin, and with a nominal volume of 2 mL. For subsequent measurements on the XN 9000/9100 (central laboratory), the remaining blood was transferred to S-Monovettes containing EDTA (Sarstedt, volume 1.6 mL).

### 2.2. Test Setup

In Group 01 (direct ctHb), original blood samples remained in the BGA cuvette after collection from the patient and were analyzed directly using the POCT devices in the test laboratory, with constant rotation.

In Group 02 (high ctHb), five consecutive BGA samples from one patient were collected and combined with dry heparin, resulting in a pooled blood sample of approximately 8 mL of blood. Plasma was separated by gentle centrifugation, and a small amount was extracted via pipette and stored separately. After remixing, the resulting ctHb was increased (Group 02, high). Measurements for this group were then performed using a new 2 mL BGA Monovette under constant rotation.

In Group 03 (low ctHb), the plasma stored from step 02 was returned to the remaining sample, reducing ctHb to a significantly lower range (Group 03: low).

Following the completion of these POCT measurements in Groups 01–03, analysis was performed in the central laboratory with the XN 9000/9100.

### 2.3. Test Systems

The GEM 4000 and the GEM 5000 (both Werfen GmbH, Munich, Germany), as well as the ABL90 Flex plus (Radiometer GmbH, Krefeld, Germany), are state-of-the-art BGA devices based on “all-in-one” cartridges containing the sensors and solutions [10]. All three devices have integrated quality management systems. The GEM 4000 requires external calibration, whereas the GEM 5000 and the ABL90 Flex plus perform this step automatically. For ctHb measurement using the CO-oximetry modules, sample volumes of 150 μL (GEM 4000), 100 μL (GEM 5000), and 65 μL (ABL90 Flex plus) are required.

The HemoCue Hb 201+ (HemoCue AB, Ängelholm, Sweden) is a compact, user-friendly point-of-care testing device based on an optical principle and a microcuvette [10]. The microcuvette design combines pipetting, hemolysis, and a dual-wavelength (550 nm and 880 nm) optical path, which compensates for turbidity.

The XN 9000 and the XN 9100 (both Sysmex Deutschland GmbH, Norderstedt, Germany) are the latest generation of “automatic hematology analyzers” (AHAs) [10]. They provide rapid, convenient measurements of ctHb in a central laboratory, using the sodium lauryl sulfate method. A key advantage is their low blood volume requirement, making them suitable for pediatric samples. In the present study, after the completion of the POCT measurements, the remaining blood in the BGA cuvette was transferred into an S-Monovette for central laboratory analysis. Constant rotation of the samples was maintained throughout the measurement protocol.

### 2.4. Data Acquisition

**Figure 1:**
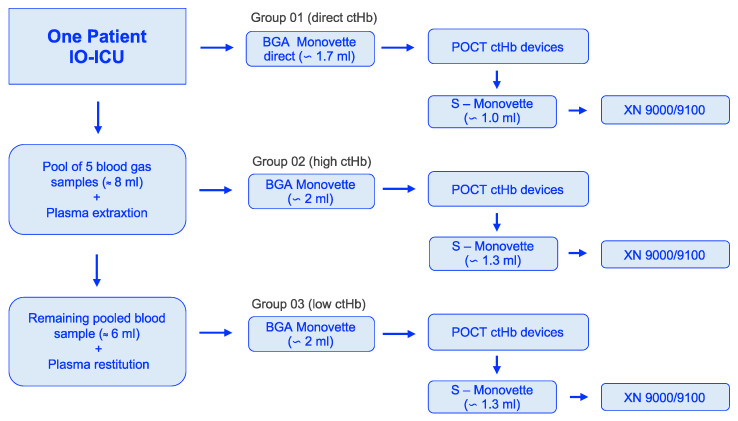
Workflow of blood sample acquisition to the selected groups: 01 (direct ctHb), 02 (high ctHb), and 03 (low ctHb). ctHb = total hemoglobin concentration; IO-ICU = interdisciplinary operative intensive care unit; POCT = point-of-care testing.

Each device received blood from a single BGA Monovette for each of the three groups (Figure 1). Therefore, one measurement value per device was included in the final evaluation, with the exception of the HemoCue 201+ system, for which three measurements were taken and averaged.

The initial bedside measurements (n = 42) identified two samples as outliers compared to the observed distribution. These outliers persisted during sample preparation to create the “high” and “low” range, distorting the structure. Consequently, n = 40 samples of N = 40 patients were available for the standardized analysis within the targeted groups.

### 2.5. Definition of References

All POCT devices evaluated here (the BGA devices and the HemoCue) and the automatic hematology analyzers XN 9000/XN 9100 can serve as reference devices for ctHb measurements (for details, see [10]). Note, however, that the HemoCue 201+ system meets reference criteria only by accepting the mean value of three measurements using three separate devices, with trained personnel, and adherence to the manufacturer’s regular quality control procedures [15]. Manual reference procedures according to DIN standards (DIN = German Institute of Standardization, Berlin, Germany, [16,17]) do not offer advantages due to inherent systematic errors introduced during processing [10,18], and these procedures involve toxic substances [19], posing an additional risk to users. Furthermore, in line with Bland and Altman’s findings, any measurement technique inherently introduces systematic error [20,21,22,23,24,25,26]. Therefore, the most consistent approach is to define the average of all test devices as the best fit reference (REF) value.

### 2.6. Statistics

The statistical analysis focused primarily on regression analyses. The root mean square error (RMSE), the mean absolute error (MAE), and the R square (RSQ) values were used to verify measurement accuracy and comparability [10].

The well-established procedures, according to Bland and Altman (B&A: bias, precision, and limits of agreement), facilitate the reproducibility of findings in this area [20,21,22,23,24,25,26].

The PI estimates the range within which a measured ctHb value will fall with a 95% probability, based on the study data [11,13].

Tolerance level analysis (TLA) was introduced [12], in contrast to Clark’s error grid representation [27], considering potential systematic errors regarding the measurement methods, with a particular focus on ctHb values < 6 g/dL [10].

## 3. Results

Blood samples from 40 patients in the IO-ICU were included in the evaluations. In addition to the primary direct and single measurement from the BGA cuvette at the patient’s bedside (Group 01 = direct), further samples from each patient were carefully processed to generate Group 02 (high) and Group 03 (low) profiles, effectively addressing the study objectives (Table 1).

**Figure 2:**
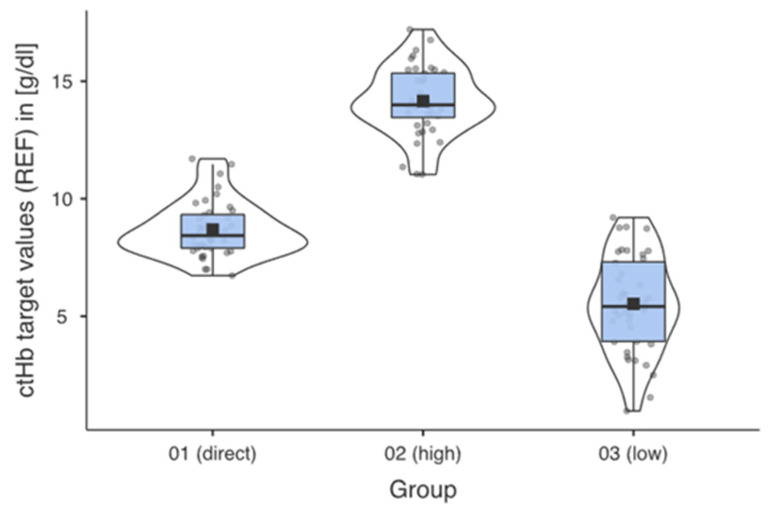
Descriptive data profiles for each measurement group with mean, median, and distribution of the measured values within the individual groups.

The ctHb ranges [g/dL] for the respective groups were the result of the systematic preparation of the samples. Figure 2 shows the successful implementation for the predefined groups.

**Figure 3:**
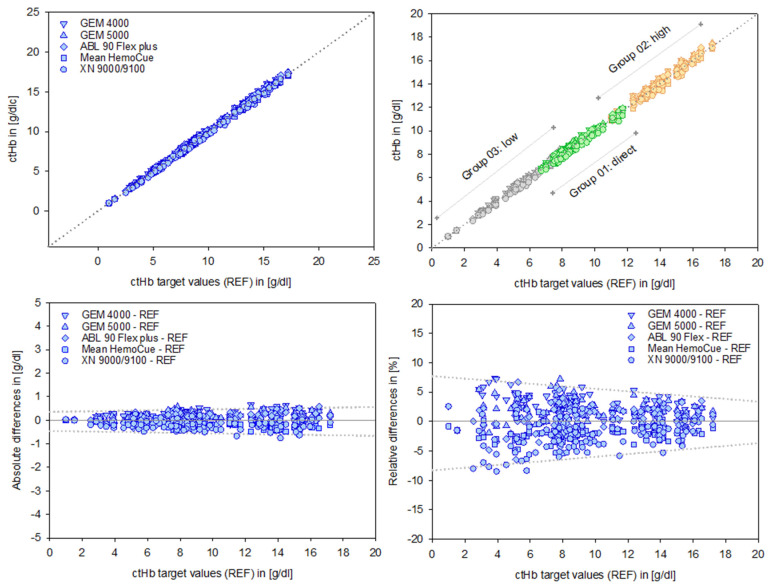
Data points of the test devices versus the ctHb target values (REF) in [g/dL], shown in the top left panel. Group-specific scales for Groups 01–03 are displayed in the top right panel. Absolute differences in [g/dL] and relative differences in [%] for the devices are shown in the bottom left and right panels, respectively. The dotted lines illustrate the course of the prediction intervals; further details are provided in the text and subsequent figures.

The alignment of the data sets, as evidenced by the regression analysis parameters (intercept and slope), indicates a high degree of consistency. The associated independent quality metrics assigned to each test system underline the robustness and reliability of these results (Table 2, Figure 3, top).

B&A plots (Figure 3, bottom) illustrate the differences between measurements, providing a precise analysis of systematic deviations. These deviations appear moderate in absolute terms [g/dL]. However, relative differences reveal the true magnitude dimensions of the deviations, considering the relevance of the initial output values. This is particularly important for low ctHb values, given their clinical relevance in this sensitive area.

A preliminary indication of the value of a PI is illustrated graphically (dotted lines, Figure 3, bottom right).

The B&A analysis in Table 3, as a basic comparison procedure for medical laboratory devices, differentiates in detail the results of the test systems as well as those related to Groups 01–03. It provides continuity with previous study performances while offering further clarification regarding data consistency. It should be emphasized that B&A comparisons inherently reference past or present data.

The total number of data pairs for the B&A analysis was n = 120, based on samples of N = 40 patients and n = 40 measurements in each of the three groups. Slight deviations in the stated number of measurement data (n = 3) for the GEM 4000 and GEM 5000 devices are due to the systems not displaying ctHb values < 3 g/dL. Additionally, a small deviation in the number of measurement data (n = 17) for the HemoCue system resulted from a temporary interruption in cuvette delivery.

**Figure 4:**
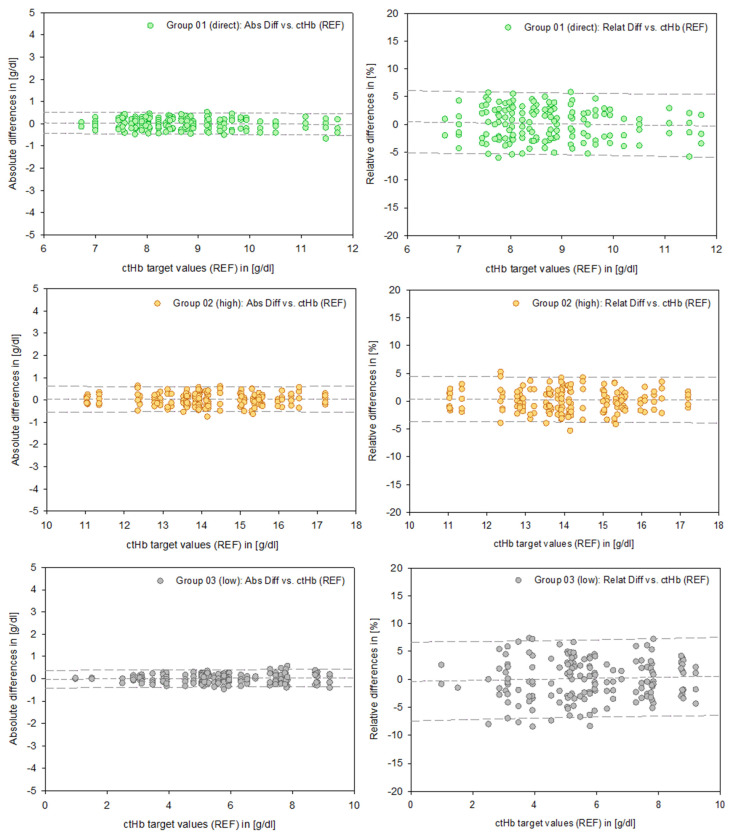
Data points of the absolute differences (**left** column, [g/dL]) and relative differences (**right** column, [%]) for Groups 01–03. The dashed lines represent the mean and the 95% prediction intervals.

In order to provide representative data regarding PIs, a differentiated reduction in the data based on the specific test systems was not applied. Therefore, Figure 4 presents uniform data across Groups 01 (direct), 02 (high), and 03 (low). This also applies to the mathematical calculation of the PIs. Consequently, the number of measurements (n) represents aggregated data across all test systems.

In contrast to this graphical representation, the PIs in Table 4 are based on a mathematical calculation referring to defined ctHb values at 2 g/dL intervals. This numerical approach allows for the straightforward clinical interpretation of a measured value within clearly defined upper and lower limits.

The upper and lower limits of the PIs for ctHb in Group 02 “high” are approximately +/− 4% or +/− 0.6 g/dL, in Group 01 “direct” are approximately +/− 4.8% or +/− 0.57 g/dL, and for ctHb in Group 03 “low” are approx. +/− 7% or +/− 0.38 g/dL, each based on relative and absolute differences.

**Figure 5:**
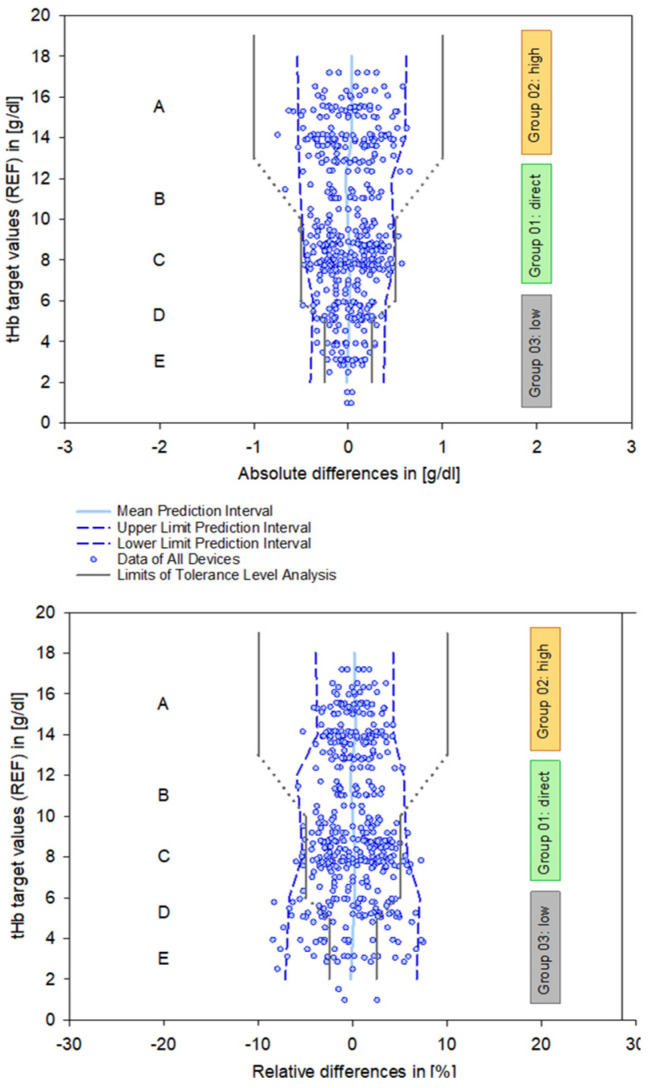
Combined data plots summarizing all devices, showing the calculated mean and 95% prediction intervals (blue lines), as well as the defined requirements of the tolerance level analysis with respect to zones A–E (solid and dashed gray lines). Zone A represents the range for anemia detection, zone C relates to patient blood management, and zone E marks the critically low measurement area. Zones B and D indicate transitional areas between these zones. Definitions according to references [10,12].

The principle of PIs is less precise than that of CIs because they provide a form of extrapolation based on a single data point. Therefore, these results need to be viewed critically when applied to clinical requirements and can be enhanced by integration into a tolerance level analysis based on clinical and regulatory limits [12]. This explicitly addresses systematic errors inherent in measurement systems, particularly in the critical clinical range below 6 g/dL (Figure 5).

## 4. Discussion

The present strictly clinical study, based on samples from BGA Monovettes from patients in the IO-ICU, demonstrates excellent agreement of the tested methods with the defined reference profile for ctHb measurement accuracy. This is particularly relevant as the samples underwent direct preanalytical processing at the IO-ICU bedside and, with the exception of the HemoCue system, were only measured once. As the ctHb spectrum of the samples obtained directly from ICU patients (Group 01: direct) did not cover the entire relevant range, additional samples were systematically processed to form “high” (Group 02) and “low” (Group 03) hemoglobin concentration groups, maintaining the same methodological conditions as the direct bedside samples. With the successful completion of the study protocol, we were able to incorporate the highly sensitive ctHb range of 1–9 g/dL, derived directly from the patients’ BGA blood samples, into the examination for the first time. The clinical importance of accurate ctHb measurement must be emphasized, as the results may require a direct and immediate therapeutic decision: the measurement accuracy of the systems is critically dependent on the baseline ctHb level. Therefore, systematic deviations in the low range—both moderate absolute differences in g/dL and more elevated relative differences in %—may not meet clinical requirements. However, the clinical implications of these inaccuracies are somewhat mitigated, as therapeutic decisions should ideally be confirmed by repeated measurements on a new blood sample.

### 4.1. Methodological Considerations

The main objective was to assess standardized BGA blood samples in the direct clinical environment of an IO-ICU, thereby reflecting the authentic clinical conditions at the IO-ICU bedside. The key difference between this study and previous laboratory tests [10] is the single, bedside measurement approach using devices and procedures that are readily available in clinical settings. The widely used laboratory reference method XN 9000/9100 and the HemoCue system hold a special position here. The preanalytical processing of the sample, from the collection via the BGA cuvette to rapid measurement (turnaround time = TAT [28]) subsumes several factors that potentially amplify the relative measurement error. Furthermore, the preanalytical variability becomes increasingly relevant at extremely low ctHb values.

The setup with the pooling of blood samples after the primary direct measurement in Group 01 (direct), followed by plasma removal to create Group 02 (high) and its subsequent reconstitution for Group 03 (low), may be methodologically controversial. The sequential order of these steps is strictly necessary and cannot be altered. However, this methodological approach allowed for the systematic generation of a sufficient sample volume to comprehensively analyze the ctHb spectrum (1–18 g/dL), a task that would otherwise be challenging (if not unattainable) using only direct clinical samples. Despite methodological concerns, the resulting measurement spectrum justifies this approach.

The lower limit obtained in the present study was below the range of 3 g/dL. However, we deliberately did not exclude these values from the analysis, even though the devices from Werfen (GEM 4000 and GEM 5000) no longer directly display this value. Why? The remaining devices presented acceptable values for these data points. In addition, the relevant guidelines of the German Medical Association for Quality Assurance in Medical Laboratory Examinations specify a lower limit of 2 g/dL [27]. Furthermore, the lowest value documented in a case report for a child that is compatible with life is 1.9 g/dL [8].

### 4.2. The Need for Quality Criteria

These parameters are defined to provide an independent assessment of data quality and allow for a higher level of comparability with results from further investigations. Initially, regression analysis provided essential parameters such as slope and intercept. Root mean square error (RMSE), mean absolute error (MAE), and R square value (RSQ) confirmed data accuracy and comparability.

Tolerance level analysis (TLA) provides a framework for mapping measured ctHb data against accuracy requirements for clinical applications [10,18]. Specifically, this analysis compares pairs of measured (TEST) and target (REF) values as a difference (TEST-REF) and as a mean of all (target reference value). This allows study data—even from different sources—to be compared with clinical tolerances. Importantly, this method continuously accounts for ctHb levels in the low range. This form of analysis is analogous to the requirements for altimeters in aircraft [25, herein “Airline Analogy”]: the closer the distance to the ground, the more accurate the measured values must be. Presentation as relative differences [in %] explicitly addressess this requirement, as opposed to the absolute differences [in g/dL].

The Bland and Altman approach for analyzing systematic deviations between two devices and the limitations of this procedures have been incorporated for the last two decades [20,21,22,23,24,25,26]. However, these data represent information from the past. Considering the need for bedside data interpretation support and the prospect that this will be based on self-learning algorithms [13], alternative approaches should be considered. PIs offer a first step toward this goal as a practical compromise suitable for this period of change [11].

### 4.3. Moving Forward from the Past to the Future

The array of test systems used here corresponds to reference standards commonly used in clinical research. In this context, measurement accuracy considerations primarily influence two key assessment approaches.

The first is the form of direct calibration already used, for example, in pulse oximetry and ctHb (further details in [29,30,31]).

The second is the establishment of a comparative reference format to evaluate emerging measurement technologies.

Regarding the development of the future generation of sensors and monitoring (point-of-care monitoring = POCM), the following techniques need to be considered:

01 Continuous and non-invasive measuring procedures [31,32,33].
02 Calculations for the estimation of blood loss [34], including online calculator tools.
03 Smartphone-based diagnostic screening technologies [35,36,37].

The overarching system that encompasses all of this is the application of machine learning techniques, which are ideal for dealing with large amounts of data and indirect variables that cannot be physically proven. A key example is the emerging use of smartphones to monitor physiological parameters such as blood pressure, ctHb [38], or glucose [39]. This can also be classified as a so-called “edge system”, which is in use in both clinical and wellness areas.

The term “prediction” thus takes on a double meaning: first, as a statistical measure represented by PIs, and second, as prognostic estimates generated by artificial intelligence from extensive data sets. Thus, the PI serves as a connecting link between past and future generations, providing a framework for more realistic and accurate predictions.

## 5. Conclusions

The immediate objectives of this study—to evaluate the ctHb measurement accuracy of clinically available devices under authentic IO-ICU conditions, to specifically analyze performance at critically low ctHb levels, and to calculate PIs to estimate future measurement uncertainty—were successfully met.

Overall, the measured values of the tested devices showed a high degree of agreement, as indicated by minimal absolute deviations. However, clinically relevant deviations occurred in relative measurements expressed as percentage differences from the baseline values, occasionally exceeding acceptable limits.

These findings provide essential information regarding the measurement accuracy of clinically relevant ctHb devices and must be carefully considered in clinical decision-making. Particular caution is warranted when interpreting ctHb values in the critically low range (1–9 g/dL), where accuracy limitations may affect critical therapeutic decisions.

It is important to highlight that PIs provide a more comprehensive statistical framework than CIs, emphasizing the clinical relevance of observed measurement deviations. However, significant uncertainties remain in clinical practice, particularly due to preanalytical factors. Therefore, it is essential to verify the measured values through repeated sampling and measuring before making therapeutic decisions.

## Figures and Tables

**Table 1:** Descriptive statistical parameters of groups 01–03. (SD = standard deviation; CI = confidence interval; N = number of patients; n = number of measurements per group).

	Group 01 (Direct)	Group 02 (High)	Group 03 (Low)
N =	40	40	40
n =	40	40	40
Mean	8.69	14.1	5.52
Median	8.43	14	5.38
SD	1.18	1.44	2.06
Minimum	6.73	11	0.975
Maximum	11.7	17.2	9.2
Shapiro-Wilk p	0.057	0.557	0.552
95% CI (lower limit)	8.33	13.7	4.88
95% CI (upper limit)	9.06	14.6	6.16

**Table 2:** Quality criteria of the data and regression analysis for ctHb in [g/dL], assigned to the test systems (RMSE = root mean square error; MAE = mean absolute error; RSQ = R square). The mean value for HemoCue is based on three measurements using identical devices.

Devices	Intercept	Slope	RMSE	MAE	RSQ
GEM 4000	0.160	1.006	0.270	0.230	0.998
GEM 5000	0.072	1.011	0.235	0.190	0.998
ABL 90 Flex plus	−0.064	1.016	0.204	0.164	0.998
Mean HemoCue	−0.053	0.985	0.229	0.203	0.999
XN 9000/9100	−0.145	0.993	0.277	0.233	0.998

**Table 3:** Data analysis according to the Bland and Altman method, including bias (mean), precision (+/− SD), and limits of agreement (+/− 1.96 SD). Absolute differences [g/dL] and relative differences [%] are reported in comparison to the respective ctHb target values (REF) [g/dL] (SD = standard deviation).

		Absolute Differences in [g/dL]				Relative Differences in [%]			
All Data		n	Mean	SD	1.96 SD	n	Mean	SD	1.96 SD
GEM 4000-REF		117	0.22	0.15	0.30	117	2.59	1.88	3.69
GEM 5000-REF		117	0.17	0.15	0.30	117	1.90	1.65	3.23
ABL 90 Flex plus-REF		120	0.08	0.19	0.36	120	0.71	2.12	4.15
Mean HemoCue-REF		93	−0.20	0.12	0.23	93	−2.19	1.11	2.17
XN 9000/9100-REF		120	−0.21	0.18	0.35	120	−2.53	2.39	4.68
Group 01 (direct)									
GEM 4000-REF		40	0.22	0.15	0.29	40	2.61	1.72	3.38
GEM 5000-REF		40	0.16	0.13	0.25	40	1.82	1.48	2.91
ABL 90 Flex plus-REF		40	0.07	0.17	0.33	40	0.82	2.00	3.92
Mean HemoCue-REF		27	−0.22	0.06	0.12	27	−2.58	0.83	1.62
XN 9000/9100-REF		40	−0.25	0.17	0.34	40	−2.87	1.84	3.61
Group 02 (high)									
GEM 4000-REF		40	0.25	0.19	0.37	40	1.79	1.38	2.70
GEM 5000-REF		40	0.22	0.18	0.34	40	1.55	1.26	2.47
ABL 90 Flex plus-REF		40	0.16	0.20	0.40	40	1.10	1.40	2.75
Mean HemoCue-REF		32	−0.27	0.13	0.26	32	−1.89	0.93	1.83
XN 9000/9100-REF		40	−0.23	0.18	0.36	40	−1.62	1.26	2.47
Group 03 (low)									
GEM 4000-REF		37	0.18	0.10	0.19	37	3.42	2.13	4.18
GEM 5000-REF		37	0.14	0.14	0.27	37	2.38	2.03	3.98
ABL 90 Flex plus-REF		40	0.02	0.16	0.31	40	0.21	2.66	5.22
Mean HemoCue-REF		34	−0.12	0.09	0.18	34	−2.16	1.33	2.62
XN 9000/9100-REF		40	−0.16	0.17	0.33	40	−3.10	3.29	6.45

**Table 4:** The 95% prediction intervals assigned to defined ctHb target values (REF) in [g/dL]. The number of data points (n) refers to the combined total of all test systems.

Prediction Intervals		Group 02			Group 01			Group 03		
		(high; n = 192)			(direct; n = 187)			(low; n = 188)		
ctHb target values (REF) [g/dL]		18	16	14	12	10	8	6	4	2
Absolute differences [g/dL]	Upper limit	0.62	0.61	0.61	0.46	0.48	0.50	0.40	0.39	0.38
	Mean	0.04	0.04	0.04	−0.03	−0.01	0.02	0.01	0.00	−0.01
	Lower limit	−0.54	−0.53	−0.53	−0.52	−0.49	−0.46	−0.37	−0.38	−0.40
Relative differences [%]	Upper limit	4.27	4.27	4.30	5.39	5.54	5.78	7.08	6.91	6.76
	Mean	0.18	0.23	0.28	−0.27	−0.02	0.22	0.17	−0.02	−0.21
	Lower limit	−3.92	−3.81	−3.75	−5.93	−5.59	−5.33	−6.75	−6.95	−7.18

## Data Availability

No further archives supporting the data available.

## Abbreviations

AHA	Automatic hematology analyzer
B&A	Bland and Altman
BGA	Blood gas analysis
CI	Confidence interval
ctHb	Total hemoglobin concentration
DIN	German Institute for Standardization
ICU	Operative intensive care unit
IO-ICU	Interdisciplinary operative intensive care unit
MAE	Mean absolute error
N	Number of patients
n	Number of measurements/data points
PI	Prediction interval
POCM	Point-of-care monitoring
POCT	Point-of-care testing
REF	Reference
RMSE	Root mean square error
RSQ	R square
SD	Standard deviation
TAT	Turnaround time
TLA	Tolerance level analysis

## References

[1] Treml B., Kleinsasser A., Knotzer J., Breitkopf R., Velik-Salchner C., Rajsic S. (2023). Hemorrhagic Shock: Blood Marker Sequencing and Pulmonary Gas Exchange. Diagnostics.

[2] Figueiredo S., Taconet C., Harrois A., Hamada S., Gauss T., Raux M., Duranteau J., The Traumabase Group (2018). How useful are hemoglobin concentration and its variations to predict significant hemorrhage in the early phase of trauma? A multicentric cohort study. Ann. Intensiv. Care.

[3] Karakochuk C.D., Hess S.Y., Moorthy D., Namaste S., Parker M.E., Rappaport A.I., Wegmüller R., Dary O., the HEmoglobin MEasurement (HEME) Working Group (2019). Measurement and interpretation of hemoglobin concentration in clinical and field settings: A narrative review. Ann. N. Y. Acad. Sci..

[4] Kawai Y., Fukushima H., Asai H., Takano K., Okuda A., Tada Y., Maegawa N., Bolstad F. (2021). Significance of initial hemoglobin levels in severe trauma patients without prehospital fluid administration: A single-center study in Japan. Trauma. Surg. Acute Care Open.

[5] Gutierrez G., Reines H.D., Wulf-Gutierrez E.M. (2004). Clinical review: Hemorrhagic shock. Crit. Care.

[6] Parish M., Abedini N., Mahmoodpoor A., Gojazadeh M., Farzin H., Sadigi S. (2017). The Association between Hemoglobin Value and Estimation of Amount of Intraoperative Blood Loss. Open J. Intern. Med..

[7] Zajak J., Páral J., Sirový M., Odložilová Š., Vinklerová K., Lochman P., Čečka F. (2024). Blood loss quantification during major abdominal surgery: Prospective observational cohort study. BMC Surg..

[8] Parodi E., Riboldi L., Ramenghi U. (2021). Hemoglobin life-threatening value (1.9 g/dL) in good general condition: A pediatric case-report. Ital. J. Pediatr..

[9] Charpentier E., Looten V., Fahlgren B., Barna A., Guillevin L. (2016). Meta-analytic estimation of measurement variability and assessment of its impact on decision-making: The case of perioperative haemoglobin concentration monitoring. BMC Med. Res. Methodol..

[10] Stawschenko E., Schaller T., Kern B., Bode B., Dörries F., Kusche-Vihrog K., Gehring H., Wegerich P. (2022). Current Status of Measurement Accuracy for Total Hemoglobin Concentration in the Clinical Context. Biosensors.

[11] Colman R. Prediction Interval, the Wider Sister of Confidence Interval. https://datascienceplus.com/prediction-interval-the-wider-sister-of-confidence-interval/.

[12] Dietzel F., Dieterich P., Dörries F., Gehring H., Wegerich P. (2019). Invasive and non-invasive point-of-care testing and point-of-care monitoring of the hemoglobin concentration in human blood—How accurate are the data?. Biomed. Eng./Biomed. Tech..

[13] Pearce T., Zaki M., Brintrup A., Neely A. High-Quality Prediction Intervals for Deep Learning: A Distribution-Free, Ensembled Approach. Proceedings of the 35th International Conference on Machine Learning.

[14] Coskun A. (2024). Prediction interval. Biochem. Medica.

[15] Sanchis-Gomar F., Cortell-Ballester J., Pareja-Galeano H., Banfi G., Lippi G. (2013). Hemoglobin Point-of-Care Testing: The HemoCue System. JALA J. Assoc. Lab. Autom..

[16] (2010). Haematology—Determination of Haemoglobin Concentration in Blood-Reference Method.

[17] (2021). Haematology—Determination of Haemoglobin Concentration in Blood-Reference Method.

[18] Gehring H., Hornberger C., Dibbelt L., Roth-Isigkeit A., Gerlach K., Schumacher J., Schmucker P. (2002). Accuracy of point-of-care-testing (POCT) for determining hemoglobin concentrations. Acta Anaesthesiol. Scand..

[19] European Chemicals Agency (2019). Candidate List of Substances of Very High Concern of the Registration, Evaluation, Authorisation and Restriction of Chemicals (REACH) Regulation.

[20] Altman D.G., Bland J.M. (1983). Measurement in Medicine: The Analysis of Method Comparison Studies. J. R. Stat. Society. Ser. D.

[21] Bland J.M., Altman D.G. (1986). Statistical methods for assessing agreement between two methods of clinical measurement. Lancet.

[22] Bland J.M., Altman D.G. (1999). Measuring agreement in method comparison studies. Stat. Methods Med. Res..

[23] Myles P., Cui J.I. (2007). Using the Bland–Altman method to measure agreement with repeated measures. Br. J. Anaesth..

[24] Abu-Arafeh A., Jordan H., Drummond G. (2016). Reporting of method comparison studies: A review of advice, an assessment of current practice, and specific suggestions for future reports. Br. J. Anaesth..

[25] Sadler A.W. (2018). Using the variance function to generalize Bland–Altman analysis. Ann. Clin. Biochem. Int. J. Biochem. Lab. Med..

[26] Gerke O. (2020). Reporting Standards for a Bland–Altman Agreement Analysis: A Review of Methodological Reviews. Diagnostics.

[27] Guideline of the German Medical Association on Quality Assurance in Medical Laboratory, Examinations; Deutsches Ärzteblatt: 2023.

[28] Rice M.J., Gravenstein N., Morey T.E. (2013). Noninvasive Hemoglobin Monitoring. Anesth. Analg..

[29] Gehring H. (2010). Point of Care Monitoring of Blood: Invasive and Non-Invasive Monitoring. European Society of Anaesthesiology, Review. https://www.semanticscholar.org/paper/Point-of-care-monitoring-of-blood-%3A-invasive-and-Gehring/4c429a47c6638a1bfeb2306f836847b0721fe005.

[30] Gehring H., Duembgen L., Peterlein M., Hagelberg S., Dibbelt L. (2007). Hemoximetry as the “gold standard”? Error assessment based on differences among identical blood gas analyzer devices of five manufacturers. Anesth. Analg..

[31] Barker S.J., Badal J.J. (2008). The measurement of dyshemoglobins and total hemoglobin by pulse oximetry. Curr. Opin. Anaesthesiol..

[32] Tang B., Yu X., Xu L., Zhu A., Zhang Y., Huang Y. (2019). Continuous noninvasive hemoglobin monitoring estimates timing for detecting anemia better than clinicians: A randomized controlled trial. BMC Anesthesiol..

[33] Man J., Zielinski M.D., Das D., Sir M.Y., Wutthisirisart P., Camazine M., Pasupathy K.S. (2022). Non-invasive Hemoglobin Measurement Predictive Analytics with Missing Data and Accuracy Improvement Using Gaussian Process and Functional Regression Model. J. Med. Syst..

[34] Hahn-Klimroth M., Loick P., Kim-Wanner S.-Z., Seifried E., Bonig H. (2021). Generation and validation of a formula to calculate hemoglobin loss on a cohort of healthy adults subjected to controlled blood loss. J. Transl. Med..

[35] Mannino R.G., Myers D.R., Tyburski E.A., Caruso C., Boudreaux J., Leong T., Clifford G.D., Lam W.A. (2018). Smartphone app for non-invasive detection of anemia using only patient-sourced photos. Nat. Commun..

[36] Zhao X., Meng L., Su H., Lv B., Lv C., Xie G., Chen Y. (2022). Deep-Learning-Based Hemoglobin Concentration Prediction and Anemia Screening Using Ultra-Wide Field Fundus Images. Front. Cell Dev. Biol..

[37] Suner S., Rayner J., Ozturan I.U., Hogan G., Meehan C.P., Chambers A.B., Baird J., Jay G.D. (2021). Prediction of anemia and estimation of hemoglobin concentration using a smartphone camera. PLoS ONE.

[38] Chen Y., Hu X., Zhu Y., Liu X., Yi B. (2024). Real-time non-invasive hemoglobin prediction using deep learning-enabled smartphone imaging. BMC Med. Inform. Decis. Mak..

[39] Rajeswari S.V.K.R., Vijayakumar P. (2024). Development of sensor system and data analytic framework for non-invasive blood glucose prediction. Sci. Rep..

